# Congenital anomalies-associated Riga–Fede disease as an early manifestation of Lesch–Nyhan syndrome: rare entities in the same pediatric patient—a case report

**DOI:** 10.1186/s12903-022-02060-1

**Published:** 2022-02-02

**Authors:** Aliaa Abdelmoniem Bedeir Eita

**Affiliations:** Faculty of Dentistry, Oral Medicine, Periodontology, Diagnosis and Radiology Department, Alexandria University, Alexandria, Egypt

**Keywords:** Riga–Fede disease, Lesch–Nyhan syndrome, Self mutilation, Rare diseases, Case report

## Abstract

**Background:**

Riga–Fede disease is a rare begnin disorder of the oral tissues, it can be associated with congenital anomalies and neurological disturbances. Lesch–Nyhan syndrome is a rare X-linked recessive disorder characterized by neurological and behavioral manifestations. A patient can rarely be diagnosed with both diseases in a lifetime. Therefore, reporting manifestations from such disorders is important to avoid misdiagnosis and help in timely intervention.

**Case presentation:**

This case report presents an 8-months-old male infant with traumatic oral ulcers from deciduous teeth. A diagnosis of Riga–Fede disease was made. Teeth grinding was performed and the oral lesions were healed. At the age of 2.5 years, the patient presented with neurological manifestations as well as facial tissue and premature teeth loss from self mutilation. Genetic sequencing revealed a variant of uncertain significance in the Hypoxanthine Phosphoribosyltransferase 1 gene. He was diagnosed with Lesch–Nyhan syndrome. Cleft palate, ventricular septal defect, congenitally undescended testis and ectopic left iliac kidney were also reported. The patient was scheduled on psychiatric treatment and after about six months of follow-up, both the behavioral and neurological symptoms were improved.

**Conclusions:**

Riga–Fede disease can be an early manifestation of Lesch–Nyhan syndrome. To the best of our knowledge, this is the first reported case with the incidence of all the mentioned entities in one pediatric patient.

## Background

Riga–Fede disease (RFD) is a rare begnin pediatric disorder that affects the oral mucosa [1]. It has a male predilection and manifests as traumatic ulcers that mainly appear in the tip of the tongue, its ventral surface and the lower labial mucosa, however, other intraoral sites can also be involved [1, 2]. RFD is the result of repetitive tongue or lip movements against natal, neonatal and primary teeth mainly the lower anteriors [1, 3].

Ulcers can on occasions cause pain that manifests as difficulties with breast feeding. The main aim of therapy is to eliminate or reduce the sources of trauma. Misdiagnosis and improper treatment could lead to dehydration, nutritional deficiencies and tissue deformity in affected infants [1, 2]. Diagnosis of RFD mainly relies on history and clinical examination. Differential diagnosis of lesions includes ulcerative candidiasis, syphilis, tuberculosis and lymphomas [4].

Riga–Fede disease can occur in infants with congenital anomalies. Also, it was found to be associated with some neuro-developmental disorders as Tourette's syndrome, congenital indifference to pain, familial dysautonomia and Lesch–Nyhan syndrome [4].

Lesch–Nyhan syndrome (LNS) is a rare X-linked recessive disorder caused by a mutation in the encoding gene for hypoxanthine–guanine phosphoribosyltransferase (HGPRT) enzyme synthesis. This results in downregulated metabolism of purine that forms the building blocks of DNA and RNA. The incidence of LNS is 1 in every 380,000 live births with almost exclusively males being affected [5].

The first manifestations of LNS can appear from a few months after birth to the teens. These can vary from elevated uric acid levels in all body fluids (hyperuricemia), neurological symptoms (hypotonia, deep tendon reflexes, involuntary body movements, developmental delay and others), intellectual disability and behavioral disturbances predominantly self mutilation which in almost all cases lead to tissue loss (including perioral and oral tissues) [6].

Diagnosis of LNS is based mainly on history, clinical examination and blood analysis. Genetic analysis to detect the mutated gene is confirmatory [6]. The target of LNS treatment is to reduce the severity of symptoms, however, the rarity of such disorder renders pediatricians often fail to diagnose and subsequently treat affected patients [7].

The aim of this article is to document a rare case of Lesch–Nyhan syndrome in a 2.5-years-old male child that showed its earliest manifestations at 8 months of age as Riga–Fede disease associated with Cleft palate, ventricular septal defect (VSD) and congenital undescended testis (cryptorchidism). Ectopic left iliac kidney was discovered later. To the best of our knowledge, this is the first reported case in the literature that presents the incidence of all such entities in the same pediatric patient.

## Case presentation

An 8-months-old male child was referred by a general dental practitioner to the oral medicine and diagnosis outpatient clinic of the Oral Medicine, Periodontology, Diagnosis and Radiology department, Faculty of Dentistry in March 2019. He was suffering from oral ulcers and difficulty with breastfeeding. The condition was concerning to his parents for which they sought medical consultation.

**Fig. 1:**
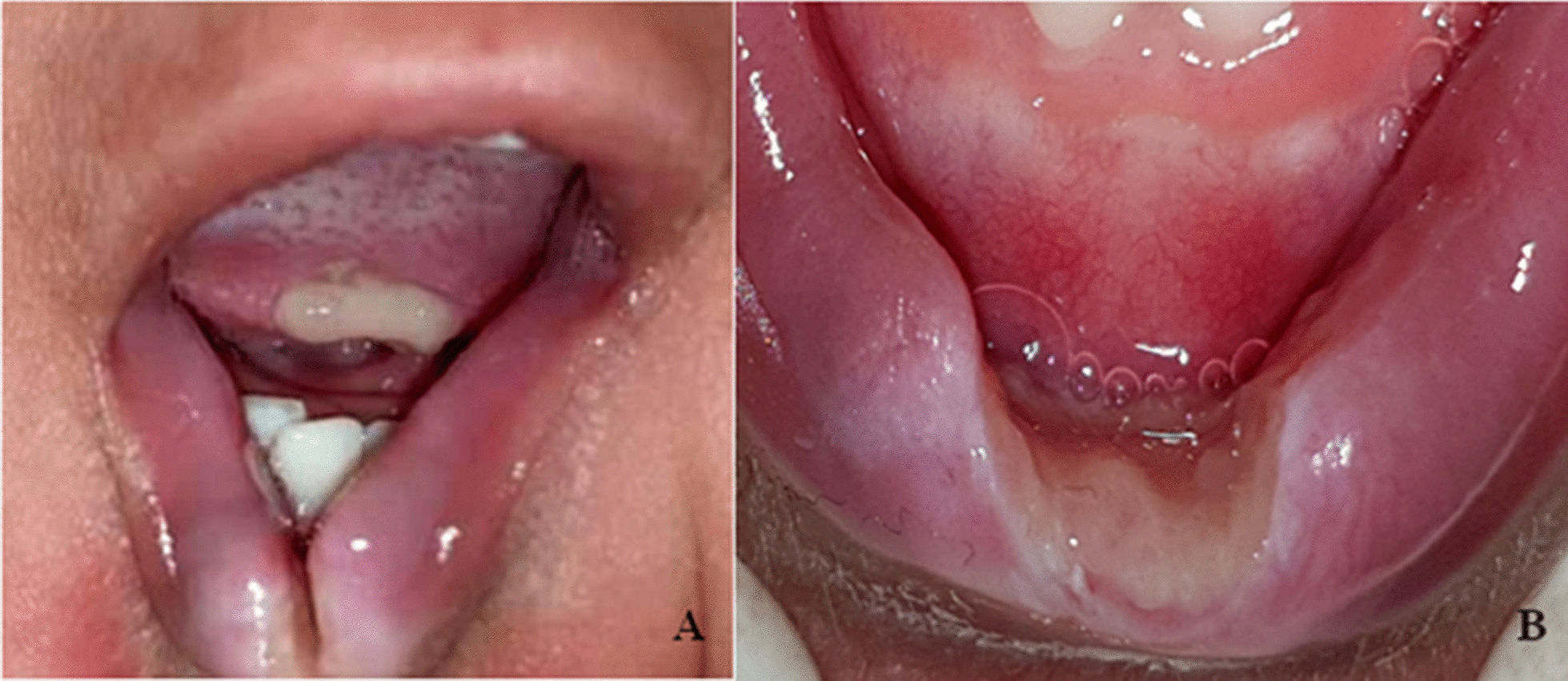
Clinical presentation of the patient at eight months of age. **A** An ulcer involving the tip of the tongue with well-defined regular borders and a yellowish central membrane. The lower lip exhibits deformity. Erythematous patches in the perioral skin is noted. **B** A deformed lower lip with an extensive ulcer involving both the oral mucosa and vermillion lip border. The ulcer has well defined regular borders and a central yellowish membrane. A white halo surrounds the ulcer borders indicating hyperkeratosis

Intraoral examination (Fig. 1) revealed two ulcers in the tip of the tongue and lower labial mucosa respectively. The labial mucosal lesion was extensive and the lower lip was deformed. Extraoral examination showed erythema of the perioral skin surrounding the lower lip. Upper and lower primary central and lateral incisors were fully erupted. The patients' mother reported the appearance of lesions two months earlier without improvement. He didn’t receive any previous medications for his condition.

The infant was born with incomplete cleft palate, VSD and cryptorchydism. Surgical correction of cleft palate was performed after birth. VSD was monitored for spontaneous closure and cryptorchydism was scheduled for surgical management. Routine laboratory investigations (CBC, coagulation profile, kidney and liver function tests) were normal. Continuous lip sucking and tongue thrusting movements ever since birth were reported. The patient had no family history.

Based on the history and clinical examination, a diagnosis of Riga–Fede disease was made for the oral lesions. Perioral dermatitis from oral habits was also reported.

A three times daily regimen of a topical analgesic (Lidocaine Hydrochloride) was prescribed along with a prophylactic topical antifungal (2% Miconazole oral gel). Also, fluid intake and vitamin supplements were recommended as supportive measures. The patient was afterwards referred to a children’s hospital. After examination by a pedodontist and consultation about the medical history by a pediatrician, grinding of primary teeth was performed to eliminate tissue trauma from oral habits. Follow up appointments were scheduled at the oral medicine clinic of the Faculty of Dentistry every week for the following month to monitor the improvement of oral lesions. Parents were incompliant with the set visits. However, follow up by phone calls was performed and the parents reported complete resolution of oral ulcers and improvement of breastfeeding after one month with persistent lip deformity. Several attempts to contact the family for further follow up were tried but they weren't reachable.

**Fig. 2:**
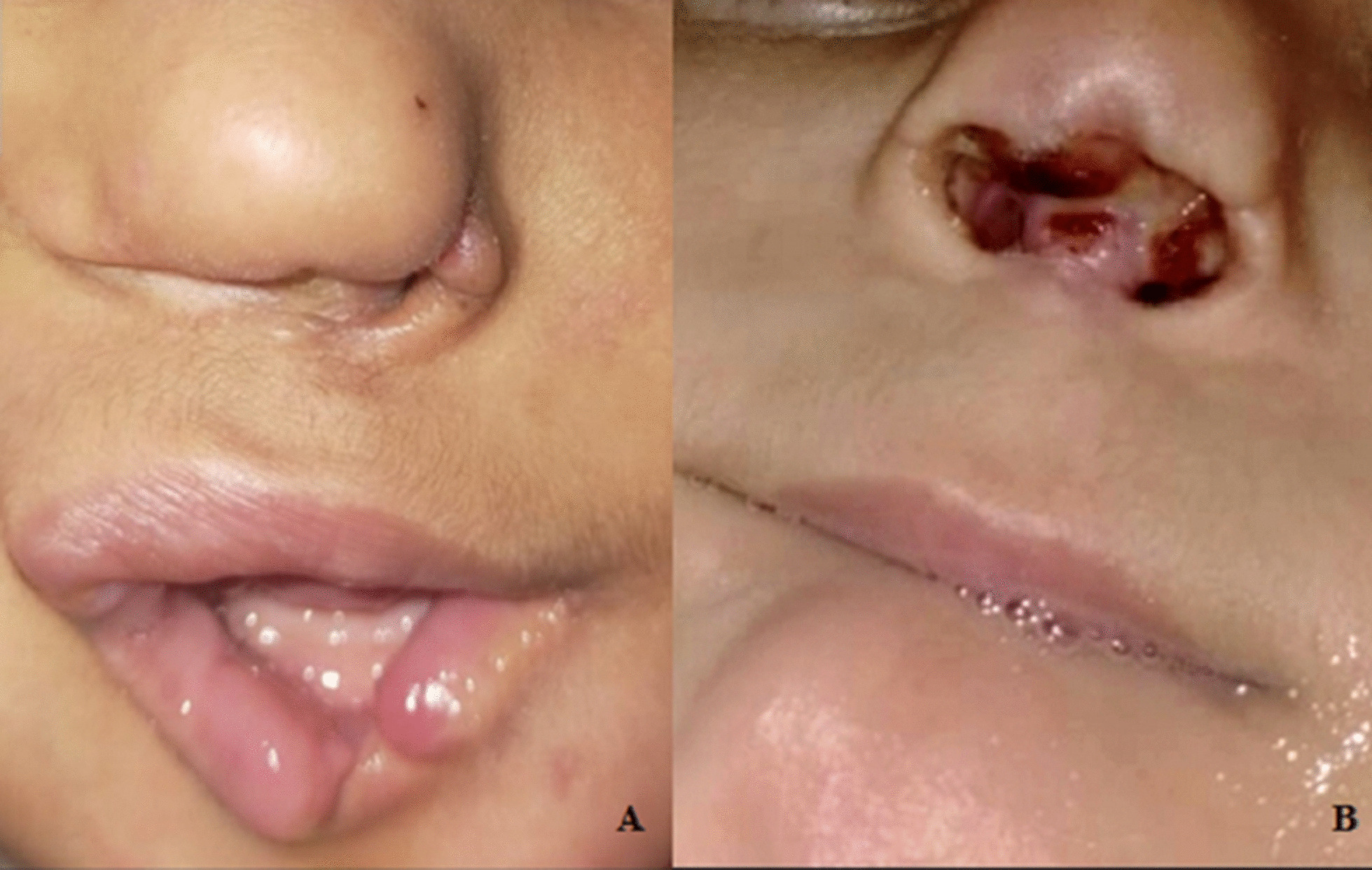
Extraoral examination of the patient at the age of 2.5 years. **A** Deformed left nasal ala and lower lip. **B** Evidence of a nasal ulcer with complete loss of the columella. Lip manipulation (sucking/biting) and saliva over the perioral tissues are evident

In January 2021, the child's parents were successfully re-contacted by the oral medicine and diagnosis clinic. Follow up revealed that the child (2.5 years) suffered from prematurely lost upper and lower primary anterior teeth for about three months. Intraoral examination revealed no oral lesions. Apart from the deformed lower lip, extraoral examination showed the presence of a nasal ulcer with total loss of the midline portion between the nostrils (columella). Furthermore, drooling was significant (Fig. 2).

The child's medical reports from the Hereditary Diseases Unit revealed the development of neurological manifestations like writhing movements of the arms and legs (dystonia), purposeless repetitive movements (chorea) and poorly articulated speech (dysarthria), as well as behavioral disturbances like aggressiveness and self mutilation. Self inflicting behaviors included head banging against hard objects, lip biting, vigorous scratching and manipulation of the eyes, nose and mouth. An eye ulcer that showed up for a few weeks before healing was also reported. Manifestations appeared earlier at the age of 1.5 years. However, pediatricians failed to reach a definitive diagnosis ever since that time. The records also reported VSD closure and normal values for kidney function tests (Urea:25.4 mg/dl, uric acid:2.5 mg/dl and creatinine:0.7 mg/dl). Abdominal and pelvic ultrasonography showed ectopic left iliac kidney. Genetic sequencing showed a hemizygous variant of uncertain significance (HPRT1, c.486-11G>A) in the Hypoxanthine Phosphoribosyltransferase 1 (HPRT1) gene. Final comments from the Hereditary Disease Unit confirmed the diagnosis of Lesch–Nyhan syndrome.

Psychiatric treatment with carbamazepine and an antipsychotic (risperidone) was scheduled to control the neurological symptoms and self mutilation. Moreover, the parents were taught some behavioral modification techniques like continuous and differential reinforcement. Such interventions were conducted under psychiatrist's supervision. As regards oral and dental management, topical antifungals were prescribed to prevent superimposed infection from drooling. Pedodontic consultation suggested that space maintenance for the prematurely lost primary teeth could be postponed to after adequate symptoms control. Dental follow up appointments were scheduled every month to monitor case improvement and rule out oral complications from self mutilation. About 27 weeks after psychiatric treatment, the patient’s parents reported significant improvement of self mutilation and neurological manifestations. No new lesions were reported. However, self inflicting behaviors are fluctuant and the patient needs regular monitoring.

A summary of the patients' full information of care are shown as a timeline in Table 1.

**Table 1:** A summary of the patient's information organized as a timeline

The event	Timeline
First clinical presentation (reported by parents)	January 2019
First medical consultation	March 17, 2019
Referral to the oral medicine clinic, diagnosis of RFD and the start of a primary treatment plan (topical analgesic, antifungal and supportive measures)	March 21, 2019
Grinding of deciduous teeth	March 25, 2019
Complete healing of ulcers and full restoration of proper breastfeeding	April 18, 2019
The appearance of neurological manifestations and self mutilating actions	January 2020
Premature loss of primary anterior teeth	From August to October 2020
The appearance of an eye ulcer from self mutilation	November 2020
Misdiagnosis of LNS	From January 2020 to January 2021
Lab investigations (kidney function tests and abdominal ultrasound) and the diagnosis of LNS	January 8 and 13, 2021
Presentation to the oral medicine clinic with a nasal ulcer and facial tissue deformity	January 28, 2021
Treatment plan for LNS	February 4, 2021
Follow-up reporting improvement of self mutilation and neurological manifestations	August 16, 2021

## Discussion

Riga–Fede disease of the oral mucosa is described as traumatic ulcerative granuloma with stromal eosinophilia (TUGSE) that appears in early life [1]. Despite being a totally begnin condition, early diagnosis and treatment are crucial to prevent the development of complications. In this case, the diagnosis of RFD relied on the history of trauma from primary teeth during the practice of oral habits. The presence of multiple congenital anomalies was supplementary and in line with the described literature [4].

Riga–Fede disease can be self limiting. Nevertheless, various treatment options have been proposed in accordance to both lesions' severity and the source of trauma, examples include extraction of natal or neonatal teeth, grinding of primary teeth, topical analgesics and sometimes corticosteroids in addition to protective oral appliances and composite resins [1, 2, 8]. Grinding of anterior deciduous teeth and a topical obtundent were the safest and most conservative approaches for the child to avoid the side effects of corticosteroids and aspiration of a removable mouth guard as a result of dislodgement from excessive movements of oral tissues especially that the patient was too young. The topical antifungal was prophylactic to avoid superimposed infection from perioral dermatitis. The supportive measures were compensatory to difficulties with feeding properly.

Lesch–Nyhan syndrome is a very rare disorder that male infants can inherit from mutated recessive genes on X chromosomes of asymptomatic carrier mothers [9]. This is the reason for the negative family history of the patient. Regardless, genetic counseling of the mother's family can reveal more than one carrier female member.

Self mutilation is the most characteristic feature of LNS. It usually appears from 1 to 3 years of age [5] as seen in the present case (it appeared at 1.5 years). The absence of HGPRT enzyme is the cause of hyperuricemia in LNS patients. As a result, orange deposits also known as "orange sand" can sometimes be seen the diapers of infants as the first manifestation of LNS which when present can contribute to the early diagnosis and management before the progression of manifestations [10]. Unfortunately, that wasn't the scenario in the present case. Nevertheless, the patient is liable to have high levels of uric acid at an older age [6].

There is no definitive long lasting treatment for LNS. However, psychiatric therapy using carbamazepines, benzodiazepines, phenobarbiturates, haloperidol, dopamine and other antipsychotic medications can help in controlling neurological symptoms [11, 12]. Behavioral modification protocols have also proven positive effect in some cases [7]. From the dental point of view, oral appliances with various designs have been constructed for controlling self injury from LNS with conflicting outcomes as regards effectiveness, complications and patient satisfaction. Extraction of teeth is considered one effective treatment option for LNS, yet it is invasive and inconvenient from the functional and aesthetic aspects^13^. In the present case, the prematurely lost primary teeth are the negative consequence of self mutilation. Contrarily, this provides a free area from sources of oral trauma which prevents further tissue injury and loss.

In this case report, an 8-months-old infant was diagnosed with RFD associated with multiple congenital anomalies. At 1.5 years of age, neurological and behavioral disturbances developed and afterwards facial tissue loss started to manifest. LNS was misdiagnosed for about one year. The parents weren’t consistently compliant with the lengthy follow up visits that were scheduled for the patient.

## Conclusions

Congenital anomalies associated Riga–Fede disease can be the earliest manifestation of Lesch–Nyhan syndrome. Meticulous history taking, early oral examination and prolonged follow up are the key to avoid misdiagnosing rare diseases and disorders and subsequently help controlling self mutilation. Parents must be instructed about all the possible associations and complications of RFD. They should consequently be motivated about the compliance with the set visits for the prolonged follow up of RFD patients.

## Data Availability

All data generated or analyzed during this study are included in this published article.

## Abbreviations

RFD: Riga–Fede disease
LNS: Lesch–Nyhan syndrome
HGPRT: Hypoxanthine–guanine phosphoribosyltransferase
VSD: Ventricular septal defect
HPRT1: Hypoxanthine phosphoribosyltransferase 1
TUGSE: Traumatic ulcerative granuloma with stromal eosinophilia
